# Population responses of common ravens to reintroduced gray wolves

**DOI:** 10.1002/ece3.4583

**Published:** 2018-10-30

**Authors:** Lauren E. Walker, John M. Marzluff, Matthew C. Metz, Aaron J. Wirsing, L. Monika Moskal, Daniel R. Stahler, Douglas W. Smith

**Affiliations:** ^1^ Yellowstone Center for Resources Yellowstone National Park Wyoming; ^2^ College of the Environment, School of Environmental and Forest Sciences University of Washington Seattle Washington; ^3^ College of Forestry and Conservation University of Montana Missoula Montana

**Keywords:** *Canis lupus*, common raven, *Corvus corax*, gray wolf, scavenger, Yellowstone National Park

## Abstract

Top predators have cascading effects throughout the food web, but their impacts on scavenger abundance are largely unknown. Gray wolves (*Canis lupus*) provide carrion to a suite of scavenger species, including the common raven (*Corvus corax*). Ravens are wide‐ranging and intelligent omnivores that commonly take advantage of anthropogenic food resources. In areas where they overlap with wolves, however, ravens are numerous and ubiquitous scavengers of wolf‐acquired carrion. We aimed to determine whether subsidies provided through wolves are a limiting factor for raven populations in general and how the wolf reintroduction to Yellowstone National Park in 1995–1997 affected raven population abundance and distribution on the Yellowstone's Northern Range specifically. We counted ravens throughout Yellowstone's Northern Range in March from 2009 to 2017 in both human‐use areas and wolf habitat. We then used statistics related to the local wolf population and the winter weather conditions to model raven abundance during our study period and predict raven abundance on the Northern Range both before and after the wolf reintroduction. In relatively severe winters with greater snowpack, raven abundance increased in areas of human use and decreased in wolf habitat. When wolves were able to acquire more carrion, however, ravens increased in wolf habitat and decreased in areas with anthropogenic resources. Raven populations prior to the wolf reintroduction were likely more variable and heavily dependent on ungulate winter‐kill and hunter‐provided carcasses. The wolf recovery in Yellowstone helped stabilize raven populations by providing a regular food supply, regardless of winter severity. This stabilization has important implications for effective land management as wolves recolonize the west and global climate patterns change.

## 1 INTRODUCTION

Top predators are now widely recognized for the effects they can have in communities by shaping competitive relationships, regulating densities of prey and smaller predators, and triggering changes to behavior, morphology, and physiology (Estes et al., 2011; Leibold, 1996; Mills, Soule, & Doak, 1993; Paine, 1996). The impacts of large carnivores on scavengers are also likely to be important in shaping food web relationships given that scavenging links transfer more energy than predation links (Wilson & Wolkovich, 2011) and that top‐down pressure from predators is believed to dampen prey population fluctuations that would otherwise be driven by bottom‐up factors (Sala, 2006). Although recent investigations have tackled how predator identity, carcass characteristics, and weather conditions may impact scavenger diversity at kill sites (Allen, Elbroch, Wilmers, & Wittmer, 2015; Elbroch, O'Malley, Peziol, & Quigley, 2017), and scavenger use of carrion in general (Selva, Jedrzejewska, Jedrzejewski, & Wajrak, 2005; Stahler, Heinrich, & Smith, 2002), the influence of top predators on local scavenger abundance has received relatively little attention. Here, we examined the influence of gray wolf (*Canis lupus*) recovery in Yellowstone National Park (YNP) on the abundance and distribution of a major scavenger within the Greater Yellowstone Ecosystem (GYE), the common raven (*Corvus corax*).

Gray wolves (hereafter wolves), once widespread across the northern hemisphere, were extirpated from much of the contiguous United States in the early 20th century. Populations in the west recently re‐established by some natural recolonization as well as intensive reintroduction efforts in the mid‐1990s in central Idaho and in the GYE, in and around YNP (Bangs & Fritts, 1996). In the GYE today, wolves influence ungulate populations, primarily elk (*Cervus elaphus*), through both direct predation (Smith, Drummer, Murphy, Guernsey, & Evans, 2004; White & Garrott, 2005) and behavioral shifts (Fortin et al., 2005; Laundre, Hernandez, & Altendorf, 2001; Mao et al., 2005). The decline and apparent stabilization of elk populations in the Northern Range of YNP have been linked to cascading effects on species throughout the food web, including plants (summarized in Ripple & Beschta, 2012), birds (Berger, Stacey, Bellis, & Johnson, 2001; Hollenbeck & Ripple, 2008), and beavers (*Castor canadensis*; Smith & Tyers, 2008). Yellowstone’s wolves have also created carrion that supplements populations of a diverse suite of scavenger species (Wilmers, Crabtree, Smith, Murphy, & Getz, 2003; Wilmers, Stahler, Crabtree, Smith, & Getz, 2003). Instead of relying on short pulses of carrion availability via hunting season or passive winter die‐off in the late winter, these scavengers now have relatively reliable access to carrion throughout the winter in the GYE (Wilmers & Getz, 2004). Furthermore, wolves in the GYE have dampened between‐year variation in winter‐spring carrion availability (Wilmers & Getz, 2004; 2005) and, by implication, may be instrumental in mediating the effects of climate change on scavenger populations and communities (Wilmers & Getz, 2005; Wilmers & Post, 2006).

Common ravens (hereafter ravens) are large, wide‐ranging, and opportunistic predators and scavengers found throughout the western United States. In areas of human development, ravens benefit from anthropogenic subsidies of food, water, and shelter, sometimes leading to artificially heightened raven densities (Boarman, 2003; Bui, Marzluff, & Bedrosian, 2010). High raven densities can also lead to increased predation pressure on raven’s prey items, including some rare and threatened species [e.g., marbled murrelets (*Brachyramphus marmoratus*; Nelson & Hamer, 1995; Raphael, Mack, Marzluff, & Luginbuhl, 2002), desert tortoise (*Gopherus agassizii*; Boarman, 2003), and greater sage grouse (*Centrocercus urophasianus*; Bui et al., 2010; Coates & Delehanty, 2010)]. In areas away from anthropogenic resources, raven populations often rely on food subsidies provided by large predators, particularly in winter, and ravens are ubiquitous scavengers at wolf kills throughout North America (Kaczensky, Hayes, & Promberger, 2005; Stahler et al., 2002; Vucetich, Peterson, & Waite, 2004). Wolves also scavenge from carcasses they find on the landscape and did not kill themselves; in areas where wolves and ravens coexist, ravens have adapted efficient ways of locating and maximizing their time at wolf kills and other wolf‐acquired carcasses (Heinrich, 1999; Kaczensky et al., 2005; Mech, 1970; Peterson, 1977; Stahler et al., 2002). Namely, ravens are known to follow wolves directly, follow wolf tracks, and are attracted by wolf vocalizations (Heinrich, 1999; Mech, 1970; Stahler et al., 2002). They feed from wolf‐acquired carcasses in large aggregations to distract wolves and other scavengers [e.g., coyotes (*Canis latrans*), bears (*Ursus* sp.), bald eagles (*Haliaeetus leucocephalus*), golden eagles (*Aquila chrysaetos*), black‐billed magpies (*Pica hudsonia*); Wilmers, Stahler, et al., 2003] and increase their individual food share (Heinrich, 1999; Stahler et al., 2002). Ravens are also highly social and in forested landscapes share information regarding carcass locations at communal roosting sites (Marzluff, Heinrich, & Marzluff, 1996). Through these adaptations, ravens are able to enjoy almost immediate discovery of wolf‐acquired carcasses, gain access to more food, and decrease the energy they spend on foraging. Studies have suggested that ravens also benefit from greater foraging success due to reduced neophobia (i.e., fear of novel items or experiences) in the presence of wolves (Heinrich, 1988, 1999 ; Stahler et al., 2002).

In YNP, ravens are the most numerous scavengers of wolf‐acquired carrion (Stahler et al., 2002). Despite their complex relationship with wolves and the considerable attention and research the wolf reintroduction has instigated in YNP, the effects of the wolf extirpation and their subsequent return to the GYE on the local raven population have not been quantified. Unfortunately, these effects are particularly difficult to tease out because historical estimates of raven populations within the GYE are noted only anecdotally throughout the literature. For example, Stahler et al. (2002) estimated the winter raven population of Yellowstone's Northern Range to be between 60 and 120 individuals, and the population in nearby Gardiner, MT, at between 300 and 500 ravens. The 2006 Yellowstone Bird Report estimated the park‐wide summer population to include approximately 300–500 ravens and a breeding population of 100–150 nesting pairs (McEneaney, 2007). Although these estimates provide useful snapshots in time, they give neither an indication of their underlying methodology nor any insight into how avian scavenger populations have changed through time in response to fluctuations in wolf abundance within the park. The interpretation of raven population changes through time is further complicated by interactions between climate and wolf provisioning to scavengers (Wilmers & Getz, 2004; 2005). In harsh winters with greater snowpack, wolves kill more frequently (Huggard, 1993; Mech, Smith, Murphy, & MacNulty, 2001; Post, Peterson, Stenseth, & McLaren, 1999) and initial consumption of prey by wolves decreases (Mech et al., 2001; Vucetich, Vucetich, & Peterson, 2012; Wilmers, Crabtree, et al., 2003), leaving a greater proportion of carrion for scavengers. Alternative foraging opportunities for ravens are provided by hunters and other carnivores (e.g., cougars; *Puma concolor*), and those opportunities may also vary through time and space. High aggregations of carrion subsidies from seasonal hunter harvests of regional ungulate populations can influence how scavengers like ravens locate and integrate anthropogenic food resources (Wilmers, Stahler, et al., 2003). While cougars on the Northern Range provision carcass biomass to scavengers, cougars often kill in steep and forested terrain and conceal carcasses by caching—behaviors that tend to minimize detection and use of their kills by ravens and other avian scavengers (Allen et al., 2015; Ruth & Murphy, 2010; Ruth, Buotte, & Hornocker, in press).

We began collecting data on raven abundance on the Northern Range of YNP in 2009 to evaluate the effect of wolf reintroduction on this important scavenger. We hypothesized a positive relationship between wolf and raven populations in areas where the species overlap. However, because ravens often come into conflict with humans and resources they value (Marzluff & Angell, 2005; Marzluff & Neatherlin, 2006), we were also interested in the factors that mediated their use of wolf‐acquired carcasses versus anthropogenic foods. Accordingly, we also evaluated how winter weather would influence raven presence in both wolf habitat and areas of human use. In years of increased winter severity, we expected ravens on the Northern Range to depend heavily on wolf‐acquired carrion and that raven presence in wolf habitat would increase. In mild winters, however, we hypothesized that more ravens on the Northern Range would utilize areas with anthropogenic food sources to supplement their diet. Given the close relationship between wolves and ravens, and behavioral adaptations of cougars to minimize avian scavenging (Allen et al., 2015; Ruth & Murphy, 2010; Ruth et al., in press), we assumed any variability in cougar abundance or cougar kill rates on the Northern Range over the study period would have a negligible impact on temporal or spatial trends in raven abundance.

## 2 METHODS

### 2.1 Study area

**Figure 1:**
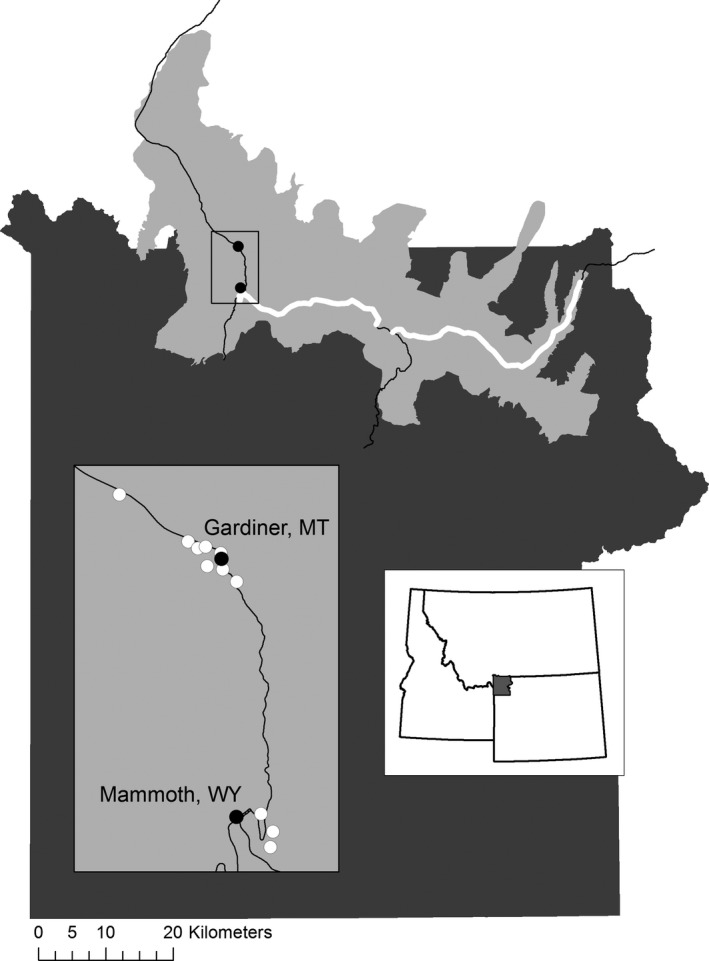
The Northern Range, shown in light gray, along the northern boundary of Yellowstone National Park (dark gray). Solid black circles are centers of human use; white circles are locations of human–area raven surveys. Roads through the Northern Range are shown; ravens were surveyed along two BBS routes (bold white lines)

The GYE, located at the confluence of Montana, Idaho, and Wyoming, comprises approximately 89,000 km^2^ of private, state, and federal land, including YNP. Within the GYE, the Northern Range is 1,500 km^2^ of grasslands in the Yellowstone and Lamar River valleys, located along the northern boundary of YNP (Figure 1).

We assessed the relationship between wolf and raven populations across the Northern Range using March observations from 2009 through 2017. Additionally, we assessed the influence of weather conditions during March on raven abundance during each survey year, including measures of total March snowfall (cm), average daily snowpack (cm), and average daily temperature (°C) in March (NOAA/National Climatic Data Center, 2017). We used weather measurements from the weather station at Mammoth Hot Springs, WY, in YNP, a location central within our study area.

### 2.2 Wolf surveys

Since the reintroduction of wolves to YNP in 1995–1997, National Park Service biologists have closely monitored and collected data on wolf predation and population dynamics in YNP. As part of this effort, YNP biologists maintain VHF and GPS collars on members of wolf packs throughout the park and, from 1996 through 2017, have monitored Northern Range wolves annually during late winter for a 30‐day study period. In most years, the study occurred entirely during the month of March. In 1997, however, the study was conducted from March 17 to April 15. During each 30‐day study, aerial crews radio‐tracked all Northern Range wolf packs daily as weather allowed (mean = 15.4 flights, range: 6–23). Additionally, ground crews intensively monitored three of those packs [hereafter “ground packs”; see Smith et al. (2004)] throughout each study period, collecting detailed information on pack movements, kills made, and carcasses acquired through scavenging. On average, ground packs were radio‐located by aerial crews on 15.1 days (range: 6–23), visually observed by aerial crews on 14.0 days (range: 3–23), and visually observed by ground crews on 21.9 days (range: 2–29) during a study period from 1996 through 2017.

#### 2.2.1 Number of wolves and wolf packs

March aerial and ground surveys allowed us to determine the number of packs on the Northern Range of YNP, as well as the number of wolves comprising each pack in each study year. We determined pack size to equal the maximum count observed on at least two occasions (Smith et al., 2004), unless that number was a poor representation of pack size (e.g., because some wolves dispersed from the pack at the beginning of a study period). To calculate the total number of Northern Range wolves, we then summed the pack sizes of all Northern Range packs within YNP. In the rare case where individual wolves were associated with two packs during a study period, we reduced the total number of Northern Range wolves appropriately to avoid double‐counting. In three cases, wolf packs spent approximately half of the March study period within our study area and we therefore included half their pack size in the total Northern Range wolf count for those study years. Because of the bias in detecting single wolves that were collared versus un‐collared, we did not include lone wolves in our estimates.

#### 2.2.2 Carcasses and biomass acquisition by Northern Range wolves

During each study period, we monitored ground packs using radio‐collared individuals and, through both aerial and ground surveys, intensively searched for ungulate carcasses that wolves fed on (i.e., either killed or scavenged; *N* = 823; Smith et al., 2004). We determined that, in most cases, wolves were the responsible predator (*N* = 724). However, wolves also scavenged five carcasses killed by cougars (*N* = 5) and also fed on 94 ungulates that did not die from predation. We estimated the live weight of each carcass as described in Metz, Smith, Vucetich, Stahler, and Peterson (2012). Following Wilmers, Crabtree, et al. (2003) and Miller et al. (2013), we then estimated edible biomass as 68% of live weight for elk, bison (*Bison bison*), and moose (*Alces alces*) and as 79% for deer and bighorn sheep (*Ovis canadensis*).

For each study period, we calculated a single annual estimate of biomass acquisition rate (biomass acquired per wolf per day; Metz et al., 2012) for Northern Range wolves. We first summed the edible biomass from carcasses acquired by the three ground packs and then divided that biomass by the total number of wolves in those three packs. We then divided this number by the average number of survey days where ground packs were located either visually by air or ground crews or, for the aircrew, using radio telemetry. Estimating biomass acquisition rate using this method reduces the effect of interannual variation in monitoring success (i.e., detecting wolves; Martin et al., 2018). Finally, to estimate the total biomass acquired by the entire Northern Range wolf population, we multiplied this annual estimate of daily biomass acquisition rate by the total number of wolves on the Northern Range.

### 2.3 Raven surveys

We monitored raven populations across Yellowstone's Northern Range during a single day during the last week of March from 2009 through 2017. To determine whether wolf presence in the Northern Range was impacting raven abundance or the distribution of the raven population, we conducted simultaneous surveys for ravens in both areas of human use and areas more closely associated with wolves.

#### 2.3.1 Ravens in human areas

To assess the relationship between ravens and humans in the Northern Range, we counted ravens in centers of human use (Figure 1) and at hunter‐killed bison carcasses. Following standard point count techniques (Ralph, Geupel, Pyle, Martin, & DeSante, 1993; Ralph, Sauer, & Droege, 1995), we conducted 11, three‐minute variable‐radius point counts for ravens in the town of Gardiner, MT, just outside the northern entrance to YNP, and at the Mammoth Hot Springs Visitor Center and nearby Employee Residence Area inside the park (Figure 1). We recorded ravens detected by sight or sound and considered the total number of ravens observed as our estimate of the raven population in human areas. We did not correct counts for variability in detection because of habitat heterogeneity and the frequent occurrence of ravens in flocks. Observers at adjacent points could have counted the same individual ravens, which travel widely and call loudly. Therefore, immediately after each count was concluded, observers compared detection times, locations, and particulars of the birds encountered to identify and remove any suspected multiple counts. Outside of Gardiner, MT, we surveyed a 1.6 km section of road where hunters process bison carcasses during a highly regulated season that typically precedes our survey. We drove the road slowly as two observers counted all ravens seen and heard on either side of the road. Upon finishing the survey in one direction, we turned and repeated the survey driving the opposite direction on the road. We added the maximum of these two road counts to the sum of ravens counted at point counts in human‐use centers to get an annual total raven abundance in human‐influenced areas.

#### 2.3.2 Ravens in wolf habitat

To assess the abundance of ravens in areas associated with wolf presence, we counted ravens along roads through the Northern Range of YNP (Figure 1) as well as at wolf‐acquired carcasses. To establish the number of ravens along Northern Range roads, we conducted surveys along two North American Breeding Bird Survey (BBS) routes (92001 and 92030), following standard BBS protocol ( https://www.pwrc.usgs.gov/bbs/participate/instructions.html; Figure 1). In the winter, roads in the Northern Range have relatively little human presence and we assumed that these surveys would be also representative of raven abundance in open areas of the park farther from roads.

We conducted ground surveys of each active wolf‐acquired carcass on the Northern Range, observing scavenger presence at the carcass over a period of several hours. Field crews scanned carcasses every five minutes and recorded the number of ravens present within the field of view. We considered the maximum number of ravens observed during these surveys to represent the number of ravens using wolf‐acquired carcasses. As in human areas, we summed ravens counted along roads and at wolf‐acquired carcasses to estimate the total number of ravens in areas associated with wolves.

### 2.4 Modeling raven abundance

To describe raven abundance across the Northern Range, we compared seven predictor variables: location (human use or wolf‐associated), three measures of weather condition (average daily snowfall in March, total March snowfall, and average daily temperature), and three measures of the impact of wolf presence during March of each study year (number of wolves, number of wolf packs, and biomass provided by wolf‐acquired carcasses). Because of a limited sample size, we compared subsets of single‐variable models to identify the best predictor variable from each of the weather and wolf variable sets. Then, in a final candidate set of models of raven abundance, we compared models with all combinations of variables that included location, the best weather and wolf variables, and two‐way interactions between location and either of the weather or wolf variables. For all models and model sets, we estimated raven abundance with generalized linear models with a Poisson error distribution. We compared models within model sets using Akaike's information criterion adjusted for small sample size (Akaike, 1974; AIC_c_) and model weights (*w_i_*). Finally, using the best model of raven abundance on the Northern Range, we predicted raven populations from 1986, ten years before the wolf reintroduction, through 2009. All analyses were conducted using the program R v 3.3.3 (R Core Team, 2017).

## 3 RESULTS

### 3.1 Northern Range weather

Between 1996 and 2017, total March snowfall averaged 21.31 cm (*SE* = 4.33; NOAA/National Climatic Data Center, 2017). The average daily snowpack during March was 9.44 cm (*SE* = 3.59), and the average daily March temperature was 1.13ºC (*SE* = 0.50; NOAA/National Climatic Data Center, 2017).

### 3.2 Wolf and raven surveys

The number of wolves and wolf packs on the Northern Range of YNP varied greatly between 1996 and 2017, peaking in the winter of 2008 with 11 packs and 92 wolves. Across all 22 years, biologists observed an average of 5.95 packs (*SE* = 0.46) and an average of 43.77 wolves (*SE* = 4.09) each March.

Between 1996 and 2017, we detected 823 ungulate carcasses acquired (i.e., killed or scavenged) by ground packs. Elk (*N* = 715) and bison (*N* = 57) comprised 94% of carcasses and deer (*N* = 20), moose (*N* = 8), and bighorn sheep (*N* = 7) made up the remainder of carcasses (16 carcasses were not identifiable to species). On average, Northern Range wolves acquired 2.11 carcasses (*SE* = 0.17), or 326.96 kg/day of prey biomass (*SE* = 28.16), per March day.

In late March of 2009 through 2017, we observed an average of 226.11 ravens (*SE* = 31.10) across the Northern Range, and the number of ravens varied substantially between survey locations (Table 1). In areas of wolf habitat, we detected an average of 107.89 ravens (*SE* = 13.21), with 54.22 ravens along roads (*SE* = 4.85) and 53.67 ravens at wolf‐acquired carcasses (*SE* = 12.35; Table 1). In human‐use areas, we detected 118.22 ravens per study year (*SE* = 31.57), including 50.22 ravens in city centers (*SE* = 9.77) and 68.00 ravens at hunter‐killed bison carcasses (*SE* = 29.46; Table 1).

**Table 1:** Ravens across the Northern Range of Yellowstone National Park during the late March survey of 2009 through 2017

Year	Wolf Habitat		Human‐Use Areas		Total
	Along Roads	Wolf Kills	Human Centers[Link]	Bison Carcasses[Link]	
2009	48	127	61	1	237
2010	37	32	49	1	119
2011	37	30	101	194	362
2012	78	77	88	29	272
2013	76	14	34	195	319
2014	53	48	12	165	278
2015	50	77	40	2	169
2016	54	12	47	18	131
2017	55	66	20	7	148

Including Gardiner, MT, as well as the visitor center and employee residential areas of Mammoth, WY.

Bison carcasses are hunter‐killed and located just outside Gardiner, MT.

### 3.3 Modeled raven abundance

Total raven abundance on the Northern Range was greatest in years with high winter snowpack (Supporting information Tables S1 and S3) and declined in years with higher March temperatures (Supporting information Table S2). Increases in raven populations also coincided with increases in the amount of prey biomass provided by wolf‐acquired carcasses (Supporting information Table S4), as well as the number of wolf packs (Supporting information Table S5) and total wolves (Supporting information Table S6) on the Northern Range during March.

The average daily snowpack in March was the best single weather predictor of raven abundance (AIC_c_ = 636.63, *w_i_* = 1.00; Supporting information Table S7). This model was substantially better than the second best model, which considered average March temperature (∆AIC_c_ = 100.91, *w_i_ *< 0.001; Supporting information Table S7). In the wolf variable model subset, the amount of carcass biomass acquired per day by wolves on the Northern Range was best at explaining raven abundance (AIC_c_ = 722.30, *w_i_* = 1.00; Supporting information Table S8). Carcass biomass was substantially better at predicting raven abundance than the second best wolf variable, the number of Northern Range wolf packs (∆AIC_c_ = 72.42, *w_i_ *< 0.001; Supporting information Table S8).

**Figure 2:**
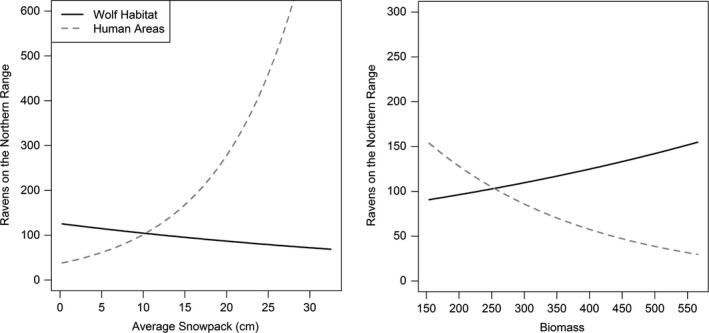
Modeled effects of average daily snowpack and amount of wolf‐acquired biomass on raven abundance in human‐use areas and in wolf habitat on the Northern Range of Yellowstone National Park from 2009 through 2017

When we evaluated the full model set, including survey location, average daily snowpack, and carcass biomass acquired per day, we found that the most complex model, considering two‐way interactions between location and each of snowpack and biomass, was best at predicting raven abundance (AIC_c_ = 379.19, *w_i_* = 1.00; Tables 2 and 3) and no other models were competitive (for the second best model: ∆AIC_c_ = 70.66, *w_i_* < 0.001; Table 2). The impact of both wolf‐acquired biomass and average snowpack on raven abundance on the Northern Range depended on the specific survey location (biomass: $\chi_{1}^{2}$ = 73.19, *p* < 0.001; snowpack: $\chi_{1}^{2}$ = 191.42, *p* < 0.001; Tables 2 and 3; Figure 2). As average daily snowfall on the Northern Range increased, raven abundance increased in human‐use areas (slope = 0.10, $\chi_{1}^{2}$ = 220.40, *p* < 0.001) and decreased in wolf habitat (slope = −0.02, $\chi_{1}^{2}$ = 12.09, *p* < 0.001; Figure 2). In contrast, as available carcass biomass increased, raven abundance increased in wolf habitat (slope = 0.001, $\chi_{1}^{2}$ = 10.52, *p* = 0.001) and decreased in human areas (slope = −0.004, $\chi_{1}^{2}$ = 69.77, *p* < 0.001; Figure 2).

**Table 2:** Models of raven abundance across the Northern Range from 2009 through 2017 considering location inside or outside Yellowstone National Park and the best variables describing weather conditions (the average snowpack per March day) and wolf presence (the biomass provided by wolf‐acquired carcasses per March day)

Model of Raven Abundance	*K*	AIC_c_	∆AIC_c_	*w_i_*
Location + Snow + Biomass + Location*Snow + Location*Biomass	6	379.19	0.00	1.00
Location + Snow + Biomass + Location*Snow	5	449.86	70.66	<0.001
Location + Snow + Location*Snow	4	458.29	79.10	<0.001
Location + Snow + Biomass + Location*Biomass	5	582.68	203.49	<0.001
Location + Snow + Biomass	4	630.72	251.53	<0.001
Location + Snow	3	635.29	256.10	<0.001
Average Snowpack	2	636.63	257.44	<0.001
Location + Biomass + Location*Biomass	4	668.82	289.63	<0.001
Location + Biomass	3	720.96	341.77	<0.001
Prey Biomass	2	722.30	343.11	<0.001
Survey Location	2	806.47	427.28	<0.001
Null	1	808.17	428.98	<0.001

**Table 3:** Parameter estimates from the top model of raven abundance on the Northern Range, considering the effects of survey location, average snowpack per March day, and the biomass provided by wolf‐acquired carcasses per March day

Parameter	Estimate	*SE*	95% Confidence Interval	
			Lower	Upper
(Intercept)	4.51	0.08	4.35	4.67
Survey Location (Human Areas)	0.19	0.11	−0.03	0.41
Average Snowpack	−0.02	0.005	−0.03	−0.008
Carcass Biomass	0.001	<0.001	0.001	0.002
Location (Human Areas):Biomass	−0.005	0.001	−0.006	−0.004
Location (Human Areas):Snowpack	0.12	0.01	0.10	0.14

**Figure 3:**
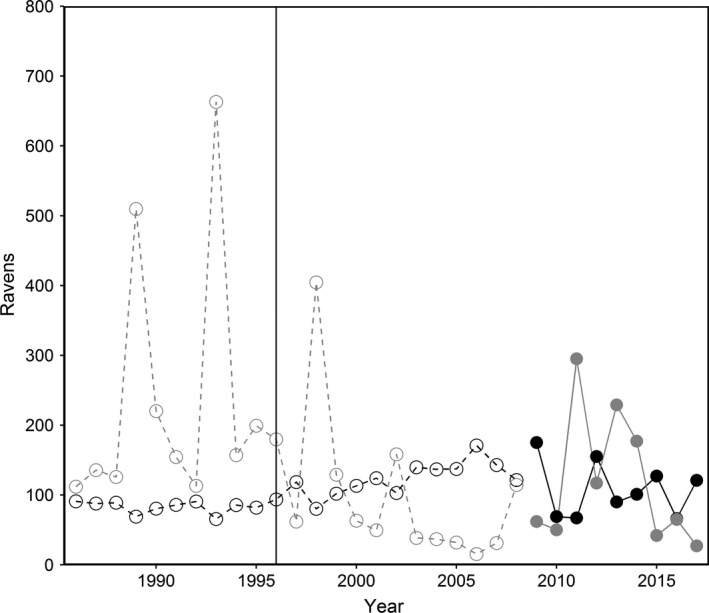
Raven abundance on the Northern Range of Yellowstone National Park from 1986 through 2017. Abundance values from 1986 to 2008 are those predicted by the top model (open points and dashed lines). From 2009 to 2017, values are ravens directly observed during surveys (solid points and lines). Black points and lines represent raven abundance in wolf habitat and gray points and lines are ravens in areas of human use. A black vertical line in 1996 represents the winter of the initial wolf reintroduction in the park

Using known values of wolf‐acquired biomass and average daily snowpack in March, we modeled raven abundance in the Northern Range from 1986 through 2008, revealing gradually increasing raven populations in wolf habitat since the 1995–1997 wolf reintroduction (Figure 3). Additionally, we found that raven populations in human‐use areas in the Northern Range were driven largely by winter snowpack and were likely highly variable before wolf populations were re‐established (Figure 3).

## 4 DISCUSSION

The relationship between predator abundance, carrion availability, and scavenger abundance has important implications for ecosystem structure and function (Wilson & Wolkovich, 2011). When predators are reintroduced to a system after being absent for an extended period of time, we may gain unique insights into the complex and cascading impacts to populations of prey and scavenger species. The return of wolves to the Northern Range of YNP has had dramatic impacts on common raven populations that are mediated by the severity of winter weather and the location of ravens within wolf habitat or in nearby centers of human use. These effects have important broadscale implications for these species across the American West, as both wolf and human populations expand and warm mild winters become increasingly common.

### 4.1 Ravens, wolves, and winter weather

Ravens are highly associated with wolves wherever their ranges overlap (Heinrich, 1999; Mech, 1970) and this pattern has held true in Yellowstone since the wolf reintroduction in 1995–1997 (Stahler et al., 2002). Ravens are more likely to be found in the vicinity of wolves than elsewhere on the landscape, especially when wolves are traveling or chasing prey, allowing these scavengers to find wolf‐acquired carcasses much faster than other carrion on the landscape (Stahler et al., 2002). By providing a reliable winter food source, wolf presence in YNP may help maintain raven populations in natural areas of the Northern Range, making them less reliant on human centers, and mediating the effects of climate change and warming winters (Wilmers & Getz, 2005; Wilmers & Post, 2006). Wolf kill rates increase in winters with increased snowpack (Huggard, 1993; Mech et al., 2001; Post et al., 1999), and we therefore expected raven presence in wolf habitat to increase in years of harsh weather. Likewise, we expected that ravens on the Northern Range would utilize anthropogenic food sources during years with mild winter weather and a decrease in wolf kill rates.

Although we found that ravens in natural areas on the Northern Range were more abundant in years when wolves provided more carcass biomass as expected, we observed the opposite of our expectations in terms of snowpack: raven abundance at anthropogenic food sources increased, and ravens in wolf habitat declined slightly, in years with greater winter snowpack. Not every carcass fed on my wolves is detected (Smith et al., 2004), and we may have therefore underestimated the total biomass that wolves provision to scavengers every March. However, variation in detection probability likely had minimal influence on our results because we standardized our annual estimates, using a method to estimate rates of biomass acquisition only on days when ground packs were actually observed (Martin et al., 2018). Furthermore, our methods were consistent throughout the study, and any underestimation of biomass would therefore be unlikely to impact the reported trends. Instead, as an explanation for our observations in years with high snowpack, we suspect that a large proportion of the raven population in the human‐use areas of the Northern Range is non‐breeding (i.e., non‐territorial) and vagrant. Most of these ravens were observed at the Gardiner, MT landfill, a year‐round food source, or at hunter‐killed bison carcasses, a predictable although temporally limited foraging opportunity. In years with mild winters, some of these non‐territorial birds may avoid competition at the landfill or bison carcasses and forage elsewhere, locating alternative food sources that may include road‐kill and possibly even wolf‐acquired carcasses. In harsh winters, however, these birds are more likely to utilize a reliable and predictable food source and point sources like the landfill likely draw ravens in from a wide radius, thus increasing total raven numbers in human‐use areas. For example, in Calgary, Alberta, ravens commuted 57.5 km to forage at a landfill and, as winter temperatures decreased and snowpack increased, raven use of the refuse site increased (Preston, 2005). Furthermore, increased bison migration outside of YNP during severe winters (Geremia et al., 2011) contributes to greater hunter‐killed bison and consequently greater access to carrion for ravens. The increase in raven abundance in human‐use areas during harsh winters was not fully compensated by the decrease in ravens in wolf habitat and instead likely reflected an influx of birds from beyond the boundaries of this survey (i.e., outside the Northern Range). In contrast to birds in human‐use areas, territorial ravens across the Northern Range are relatively familiar with the local wolf packs and are able to locate and scavenge from wolf‐acquired carcasses almost immediately (Stahler et al., 2002). Thus, although these birds may range widely in winter, they probably rely heavily on wolf‐acquired carrion regardless of winter snowpack. Although raven numbers at wolf‐acquired carcasses may decline slightly in harsh winters as some non‐territorial birds return to predictable food sources, the number of territorial Northern Range birds scavenging from wolf‐acquired carcasses remains relatively stable. Between 2009 and 2017, we observed between 12 and 127 ravens on wolf‐acquired carcasses at any one time, similar to observations of 3–135 ravens by Stahler et al. (2002) from 1997 to 2000. Thus, we believe the fluctuations in raven abundance both in wolf habitat and at human‐provisioned point sources are likely driven largely by non‐breeders.

Wolf populations are increasing and spreading across western North America, resulting in increased conflict with humans (Bangs et al., 2005). With a more thorough understanding of how wolves affect populations of other native species, we can better manage ecosystem impacts and perhaps mitigate wolf–human conflict. Furthermore, understanding how the increasing presence of wolves in western North America also influences the abundance of ravens and their use of anthropogenic resources can inform land owners and managers of possible shifts in depredation of crops, rare species, and other natural resources. Increased association of ravens with wolves throughout the western USA should benefit land managers by reducing some conflicts and increasing the efficacy of non‐lethal raven deterrents, which are increasingly viewed as morally offensive (Marzluff & Swift, 2017). Our results suggest that in areas where wolves provide abundant carrion, ravens will be less of a nuisance in nearby rural communities and agricultural settings, especially during mild weather. Those ravens that remain in these areas, which we suppose will be primarily non‐breeding, subordinate birds, may also be hazed away more easily because of alternative scavenging opportunities in the region. We are less certain how the susceptibility of rare species, such as marbled murrelet, desert tortoise, and greater sage grouse, to raven predation will respond to wolf presence. As raven abundance increases following wolf recolonization, nearby rare species may garner more incidental predation. But if ravens are sated by carrion, then predation may decline. The latter possibility would be most likely in cases where territorial adult ravens, which come to closely associate with nearby wolves (Stahler et al., 2002), are the primary predators [e.g., as with desert tortoise (Boarman, 2003) and greater sage grouse (Bui et al., 2010)]. The effect of wolf presence on raven foraging patterns may also be seasonal—raven populations boosted by wolf‐acquired carrion in the winter may place increased predation pressure on prey species during the summer.

### 4.2 Predictions of historical raven abundance

Based on our observations, we modeled raven abundance across the Northern Range to demonstrate the effects of wolf restoration on this important scavenger. Our modeled estimates of raven populations in natural areas of the Northern Range were relatively low prior to wolf reintroduction, and our results suggest more territorial, breeding pairs of ravens now utilize the Northern Range. In human‐use areas, however, we predicted raven abundance to be comparatively high and variable before the wolf population was re‐established, fluctuating with winter severity. After wolves were reintroduced, raven abundance in human areas was still predicted to be high and cyclic but fluctuations were somewhat dampened. Our models were based on relatively few observations and the predicted fluctuations in human–area raven populations are probably unrealistically dramatic, particularly prior to the wolf reintroduction. However, we expect that, even prior to the return of wolves to YNP, raven populations likely cycled with patterns in late winter hunter harvests of elk and bison, as well as winter severity, perhaps tied with the availability of winter‐kill (i.e., the passive winter die‐off of elk weakened by injury or age).

Importantly, our predictive models do not directly account for winter‐kill or hunter harvest within the study area. Winter‐kill historically provided scavengers, including ravens, with a pulse of abundant late‐winter resources; the rest of the year, however, the availability of carrion was scant and unpredictable (Gese, Ruff, & Crabtree, 1996; Houston, 1978). Furthermore, winter‐kill, even when available, would not have benefitted ravens in the same way that wolf‐acquired carrion does; ravens locate and forage from wolf‐acquired carcasses on the Northern Range almost immediately, but they can take significantly longer to find and feed from non‐wolf‐acquired carrion (Stahler et al., 2002). This delay likely results in a loss of carrion biomass to other competing scavenger species, like coyotes, bald and golden eagles, and late‐winter emerging grizzly bears. Thus, the contribution of winter‐kill to the Northern Range's raven carrying capacity was likely low.

While winter‐kill was available across the Northern Range, carrion from late‐winter hunter harvests was historically aggregated both spatially and temporally (Wilmers, Stahler, et al., 2003). From 1976 through 2009 (the first year of this study), the state of Montana issued thousands of antlerless elk tags in a special late‐season elk harvest designed to reduce the number of elk in the Northern Range herd (Lemke, Mack, & Houston, 1998). In most of these winters, 1,000–2,000 or more cow elk were harvested in the Gardiner, MT area, in January and February. Coupled with periodic bison hunts conducted by federal, state, and tribal agencies, thousands of kilograms of carrion biomass were available to scavengers in late winter months.

Additionally, following their extirpation in the 1930s, cougars naturally recolonized the Northern Range in the 1980s, increasing in abundance simultaneous to wolf recovery (Ruth, 2004; Ruth et al., in press). Due to scant hunting pressure and a reestablishing population under relatively high elk abundance, increasing cougar abundance with increasing wolf numbers indicated that cougars were not being limited by wolves. However, although cougars are an important predator of elk in the study area, they tend to kill in topographically rougher and more heavily forested terrain than wolves (Ruth et al., in press). This, coupled with cougar behavior of concealing kills by dragging them to thicker cover and caching kills under debris such as grass, hair, sticks, and snow minimizes detection and utilization by ravens (Allen et al., 2015; Ruth & Murphy, 2010; Ruth et al., in press) and likely a less important contribution to raven abundance.

### 4.3 Ravens in a changing climate

The return of wolves to YNP may help buffer populations of prey species, particularly elk, and scavengers from the effects of impending climate change (Wilmers & Getz, 2005; Wilmers & Post, 2006; Wilmers, Post, & Hastings, 2007). We found that wolves and the regular provisioning of carrion allowed for an increase in breeding raven populations in natural areas of YNP's Northern Range and dampened fluctuations in the total raven population due to winter severity. In general, larger and more stable populations are more resilient and less vulnerable to extinction (Lande, 1993). Ravens were not at immediate risk of local extinction before the wolf reintroduction and, in fact, their numbers have been increasing in the west since the late 1960s (Sauer et al., 2017). However, some predictions suggest that climatic conditions in the Rocky Mountains will become progressively less favorable for ravens in the coming decades, particularly during the breeding season (Langham, Schuetz, Distler, Soykan, & Wilsey, 2015), and the effects of climate change on ravens in YNP are largely unknown. The presence of wolves, and, consequently, a regular source of carrion, may facilitate the persistence of ravens as well as the persistence of other climate‐threatened avian scavengers, including eagles and magpies.

## CONFLICT OF INTEREST

None Declared.

## AUTHOR CONTRIBUTIONS

LW, JM, and DWS designed the study, and all authors helped organize and execute data collection in the field. MCM calculated wolf, wolf pack, and biomass variables and wrote the initial “Wolf survey” methods section. LW conducted analyses and wrote the initial version of the manuscript. JM, AW, MCM, DRS, and DWS contributed substantially to revisions.

## DATA ACCESSIBILITY

Data available from the Dryad Digital Repository: https://doi.org/10.5061/dryad.j3qt5pf.
