# Supplementary material for: Population responses of common ravens to reintroduced gray wolves
Source: Ecol Evol. 2018 Oct 30;8(22):11158–68. doi: 10.1002/ece3.4583 (PMC6262918; doi:10.1002/ece3.4583)
Supplement: Supplementary file 1 [file ECE3-8-11158-s001.docx]

**SUPPORTING INFORMATION**

Table S1. Parameter estimates from the model of raven abundance on the Northern Range considering the effects of average snowpack per March day (cm). Compared with other single weather variable models, the model considering average snowpack best explained raven abundance (∆AIC_c_ = 0.00, *w_i_* = 1.00) and improved upon the null model by a ∆AIC_c_ of 171.55 (Table S7).

|  |  |  | 95% Confidence Interval | |
| --- | --- | --- | --- | --- |
| Parameter | Estimate | SE | Lower | Upper |
| (Intercept) | 4.43 | 0.03 | 4.37 | 4.50 |
| Average Snowpack | 0.03 | 0.002 | 0.02 | 0.03 |

Table S2. Parameter estimates from the model of raven abundance on the Northern Range considering the effects of average temperature per March day (°C). Compared with other single weather variable models, the model considering temperature had a ∆AIC_c_ of 100.91 (*w_i_* < 0.001) but improved upon the null model by a ∆AIC_c_ of 70.64 (Table S7).

|  |  |  | 95% Confidence Interval | |
| --- | --- | --- | --- | --- |
| Parameter | Estimate | SE | Lower | Upper |
| (Intercept) | 4.87 | 0.03 | 4.82 | 4.92 |
| Average Temperature | -0.14 | 0.02 | -0.17 | -0.11 |

Table S3. Parameter estimates from the model of raven abundance on the Northern Range considering the effects of total March snowfall (cm). Compared with other single weather variable models, the model considering total snowfall had a ∆AIC_c_ of 107.10 (*w_i_* < 0.001) but improved upon the null model by a ∆AIC_c_ of 64.45 (Table S7).

|  |  |  | 95% Confidence Interval | |
| --- | --- | --- | --- | --- |
| Parameter | Estimate | SE | Lower | Upper |
| (Intercept) | 4.40 | 0.05 | 4.31 | 4.49 |
| Total Snowfall | 0.01 | 0.002 | 0.01 | 0.02 |

Table S4. Parameter estimates from the model of raven abundance on the Northern Range considering the effects of the amount of biomass provided by wolf-acquired carcasses per March day. Compared with other single wolf variable models, the model considering carcass biomass best explained raven abundance (∆AIC_c_ = 0.00, *w_i_* = 1.00) and improved upon the null model by a ∆AIC_c_ of 85.88 (Table S8).

|  |  |  | 95% Confidence Interval | |
| --- | --- | --- | --- | --- |
| Parameter | Estimate | SE | Lower | Upper |
| (Intercept) | 4.31 | 0.05 | 4.21 | 4.41 |
| Carcass Biomass | 0.001 | < 0.001 | 0.001 | 0.002 |

Table S5. Parameter estimates from the model of raven abundance on the Northern Range considering the effects of the number of wolf packs. Compared with other single wolf variable models, the model considering the number of wolf packs had a ∆AIC_c_ of 72.42 (*w_i_* < 0.001) and improved upon the null model by a ∆AIC_c_ of 13.45 (Table S8).

|  |  |  | 95% Confidence Interval | |
| --- | --- | --- | --- | --- |
| Parameter | Estimate | SE | Lower | Upper |
| (Intercept) | 4.40 | 0.09 | 4.23 | 4.57 |
| Number of Wolf Packs | 0.06 | 0.01 | 0.03 | 0.08 |

Table S6. Parameter estimates from the model of raven abundance on the Northern Range considering the effects of the number of wolves. Compared with other single wolf variable models, the model considering the number of wolves had a ∆AIC_c_ of 84.48 (*w_i_* < 0.001) and improved upon the null model by a ∆AIC_c_ of 1.39 (Table S8).

|  |  |  | 95% Confidence Interval | |
| --- | --- | --- | --- | --- |
| Parameter | Estimate | SE | Lower | Upper |
| (Intercept) | 4.54 | 0.10 | 4.34 | 4.73 |
| Number of Wolves | 0.005 | 0.003 | < 0.001 | 0.01 |

Table S7. Models of raven abundance across the Northern Range from 2009 through 2017 considering single variables of weather condition.

| Model of Raven Abundance | *K* | AIC_c_ | ∆AIC_c_ | *w_i_* |
| --- | --- | --- | --- | --- |
| Average Snowpack | 2 | 636.63 | 0.00 | 1.00 |
| Average Temperature | 2 | 737.54 | 100.91 | < 0.001 |
| Total Snowfall | 2 | 743.73 | 107.10 | < 0.001 |
| Null | 1 | 808.17 | 171.55 | < 0.001 |

Table S8. Models of raven abundance across the Northern Range from 2009 through 2017 considering single variables of wolf presence.

| Model of Raven Abundance | *K* | AIC_c_ | ∆AIC_c_ | *w_i_* |
| --- | --- | --- | --- | --- |
| Carcass Biomass | 2 | 722.30 | 0.00 | 1.00 |
| Number of Wolf Packs | 2 | 794.72 | 72.42 | < 0.001 |
| Number of Wolves | 2 | 806.78 | 84.48 | < 0.001 |
| Null | 1 | 808.17 | 85.88 | < 0.001 |
